# Increasing and decreasing factors of hope in infertile women with failure in infertility treatment: A phenomenology study 

**Published:** 2014-02

**Authors:** Leili Mosalanejad, Nehle Parandavar, Morteza Gholami, Sareh Abdollahifard

**Affiliations:** 1 *Deprtment of Mental Health, Jahrom University of Medical Sciences, Jahrom, Iran.*; 2 *Department of Maternity, Jahrom University of Medical Sciences, Jahrom, Iran.*; 3 *Department of Maternity, Jahrom University of Medical Sciences, Jahrom, Iran.*

**Keywords:** *Assisted reproductive technology*, *Infertility*, *Hope*, *Qualitative research*, *Phenomenology*

## Abstract

**Background:** Assisted reproductive technology (ART) provide the hope of pregnancy for infertile women, but do not always turn this hope into reality.

**Objective:** The purpose of this study was to explore the lived experience of infertile women from increasing and decreasing factors of hope in infertile women with failure in infertility treatment.

**Materials and Methods: **Using a qualitative research design (Phenomenology study), 23 subjects were selected who had experienced infertility failure visited by gynecologist (Rasekh Infertility center) in 2012. The data were collected through semi structured interviews and analyzed using interpretive research strategies of phenomenology by Collizi's seven-stage method.

**Results: **Totally 96 codes were identified. The data arranged in two categories. The factors decreasing and increasing hope in infertility treatments. Totally 5 themes and 20 sub themes were extracted. The increasing factors which emerged from the data contain "spiritual source", "family interaction and support" and "information through the media", and decreasing factors contain "nature of treatments" and "negatively oriented mind".

## Introduction

Infertility is one of the important disorders and diseases in the human community. Fertility is highly valued in most cultures (1). Infertility is defined as lack of fertility after one year of intercourse without using any kind of contraceptives (2). Statistically, 25% of the women undergo some kinds of infertility in their lives. In most participants, frequency of treated and untreated infertility is 13-19% in which untreated portion possesses 2.4-5.9% (3). Studies carried out in Iran also revealed that the prevalence of infertility in rural and urban areas was 5.3% and 6.8% respectively (4). For many women the effect of infertility and notably of medical therapy is a considerable emotional stress (5). Also it was shown that, infertile women undergo more tension, anxiety, depression, self-reproach, and suicide temptations (6). 

Losing hope to bear a child which is symbolically significant for the women carries a lot of sorrow which is intensified seeing mothers with their children (7). Hope is one of the vital psychological needs. In other words, hope makes self-confidence, internal positive feeling toward a particular thing or event. Materializing a purpose is impossible without hope and conversely, it makes people believe that darkest scenario would not happen to them (8). Hope predicts physical and mental health as determined by various indicators such as self-reporting health, positive response to medical interventions, mental health, positive mood, effective coping, reassessment, problem solving, avoiding stressful event, seeking support, and health promoting behavior (9). 

Applying Schneider theory regarding hope, it has been discussed even in the death process and the final stages of diseases. According to this strategy, maintaining and even increasing hope in medical interventions for dying patients is considerably beneficial (10). This strategy is seeking to assist workers to formulate their purposes and make several passes to reach them, stimulate themselves to follow the purposes and reframe up hindrances as challenges to overcome and hope as a sort of treatment (11). 

Some others know it to have a significant role in people’s attitudes toward their lives (8). Compared to false hope (pessimism), hope (real hope) is associated with positive health, and decreasing hope has poor results (12). Hope in infertility treatment is one of the most important factors in In vitro fertilization (IVF) achievement (13). In other word positivists, in comparison to pessimists, are less likely to suffer from poor physical health or depression and disappointment or committing suicide (14). During and after long and sometimes unsuccessful treatments impulsive behavior, anger, depression, helplessness, worthlessness, inadequacy, anxiety and negative beliefs may occur (15). 

Fertility attempts require tedious and expensive medical procedures thus, doubt and despair during treatment could threaten a couple (7). In study by Brendes *et al* the major reasons of discontinuation of treatment by IVF have been mental and affective problems (22.3%), weak prognosis of treatment (fear and hope to success in treatments (18.8%) and failure in infertility treatment (17.2%) (16). In a systematic review carried out by Gameiro *et al* the reasons of treatment discontinuation consisted of: prolong process of treatments (39.18%), unknown (19.17%), physical and mental factors (19.07%), conflict with others (16.67%), personal reasons (9.27%), communication problems (8.83%), refusal of treatment (13.23%), organizational (11.68%) and health care service (7.71%) (17). 

As mentioned before, hope creates motivation and enthusiasm in individuals to reach their goals and overcome their difficulties. Studies which encompass all aspects and dimensions of the challenges associated with the infertility treatments are few in Iran. Most of information is received from sources and media, and this is the expert view of points. There are few studies that can cover this topic and investigate them thoroughly (18). 

Also there is a qualitative study that examines the experience of infertility as a problem however, infertility treatments are not considered in any research (19-20). Due to the importance of hope as a unique experience in this type of treatment, this study is designed to investigate perceptions and experiences of infertile women with a history of failure in treatment procedure. Recognizing increasing and decreasing factors of hope in infertile women, particularly from live experiences and the viewpoints of people who deal with them can lead to present appropriate strategies in infertility treatment because they play an important role in designing treatment programs in infertile couples. 

## Materials and methods

This study was a qualitative study with a phenomenological approach (Husserl Ian descriptive phenomenology) carried out on infertile women attended to Rasekh Infertility Clinic in Jahrom in 2012 (21). Phenomenology study was used in this study. "Phenomenology study is the natural affinities to access the person’s live experience "a phenomenological study is one that focused on descriptions of what people experience and how it is that they experience what they experience" (22). Purposeful sampling was conducted on a group of women who failed in assisted-infertile treatments. 

**Data collection**

Inclusion criteria in this study were included: being native Persian, infertility diagnosed by gynecologist, referring to infertility clinic in Jahrom in 2012 with a history of at least one failure in treatment procedure, no record of chronic diseases, as well as physical and psychiatric diseases. Participants had primary infertility and were willing to participate in the interview. Sampling began purposefully by semi-structured interview. The interview lasted about 35 min for each participant ranging from 30-45 min. The questions were made by applying the statement about “when talking about infertility treatments, what is associated in your mind? What is promising for you in these infertility treatments? What factors decreases hope in you in these treatments? Further clarification regarding construction of the final questions expanded through the use of a pilot study. All available tapes would be copied and kept for further use according to data protection. In this study the researcher would applied pilot methods including confidence making in speaking, evaluating the room appropriateness, checking all equipment’s such as audio recording and so on. 

**Data analysis**

All of conversations were recorded, if not, they were rewritten word by word. The main question of this study was hope increasing and decreasing factors in individuals receiving assisted-fertile treatments. The seven-steps Colaizzi's method of data analysis was used in this study. Colaizzi's seven-step-method was employed to present a profound description of the structure of the women experience Colaizzi (1978). Colaizzi’s method has some advantages (23). This method includes:

- Reading and rereading descriptions
- Refer to the narratives frequently and extract significant statements.
- Formulating meanings (to illuminate meaning hidden in various context of the phenomena)
- Categorizing into clusters of themes and validating.
- Describing
- Returning

Incorporating any changes based on the informants’ (22).

**Methodological rigor**

Criteria for judging the rigor of such research include "credibility”, “transferability”, “dependability”, and “conformability” which are determined in resent research (24). To provide credible research, we adhered to guideline of being professional with integrity, intellectual rigor and a methodological capability (25). Transferability refers to whether findings of a study can be applied to in different context (26). We provided a rich description of experiences of participating in ART and related factor to change hope in following treatment program .Dependent ability considers whether findings of a study would be similar if the study was to be replicated (25). 

All the interviews transcript personality and to ensure that they did not contain mistakes, also we included contributions from peer debriefing (three experts in qualitative research) during the data analysis. Conformability suggests data, interpretation and findings of the research are not the creation of the research and that the data as well as the interpretations can be related to its source (27-28). After code reviewing and reaching agreement they were used. Recording all stages of the study makes it possible to transfer to the researchers. Ethical considerations in this study were content of the participants, granting permission to record interviews, respect to preservation of confidentiality and anonymity. 

During interview an attempt was made to provide privacy and convenience of the participants, and participants’ personal information was kept confidential, and they were assured that sound file would be removed after use. All of the participants were satisfied with the project and there was no coercion to participate in research. Proposal extracted from this paper confirmed by ethical committee in Jahrom University of Medical Sciences.

## Results

Of all 96 codes were identified. 23 participants were selected. Participants’ age mean was reported (28±2.6) years old. Most of participants with 66.7% had women infertility reasons and the rest had men reasons. The mean treatment duration was (7±1.6) maximally. Totally 2 had middle school education, twelve high school diploma, 5 associated degrees, and 4 had bachelor’s degrees. Eleven had undergone at least once IUI and 5 IVF treatments and others used medicine therapy to solve their infertility problems. 

All 96 extracted codes of data were investigated in two groups of hope decreasing and increasing factors which were led to categorization of themes into three increasing and two decreasing factors. The first main identified theme contains increasing factors (spiritual resources, family interaction and support and media) and the second main identified theme contains decreasing factors (nature of treatments & negatively oriented mind). Increasing factors was allocated to spiritual resources which were revealed such as:

**Saying prayer to God**

Prayers are demonstrated in the experiences of these people in various dimensions so that coming close to God can facilitate life hidden good intentions and provide motivation to follow the treatments. Another person who failed IUI says: “I resort to God then doctors. Saying prayers escalates hope in me, more than doctor’s words. A motive is created suddenly, but prayer in really effective.” Some believe that visiting holy shrines and even seeing them keep hope alive so that a person who had 3 unsuccessful IVF treatments finds donation and charity as a key to increase hope and believes: “I try to do some good works and by doing so, I feel others’ prayers give me self-satisfaction.”

**Shrines**

Shrines are situations used by people as a spiritual resource and increasing feel of hope in people. A person who failed IVF treatment says: “Some days ago it accidentally happened to me to visit Imam Reza shrine and I told myself Imam Reza would fulfill my wish since he has invited me”. 

**Family prays**

According to sayings, family prayer is rich spiritual source that gives people hope and a good sense of the aspirations." I have a grandmother who always prays for me and says: I just pray for you to bear a child. Sincere pray of family keeps hope alive that eventually the work". The second theme contain family interaction and support is the support and sympathy of others which is really effective in creating hope, attachment, and motive in treatment from the viewpoints of infertile women, and allocates subthemes such as couples' interaction ,and sympathy. 

**Couples' interaction**

Positive interaction between couples is a source of hope in many women. Woman who failed treatment and her husband is addicted says: “my husband is the source of hope for me, he is addicted. The love between us gives me hope. Although he is addicted, he loves me and it gives me hope.” Another woman who has experienced IVF cycle says: “My husband keeps telling me don’t worry; you can bear a nice girl.”

**Sympathy**

In most participants, family role is emphasized and individuals stress the role of their families particularly mothers and husbands and their promising roles. A woman who has failed IUI cycle states: “I do IUI again, it gives me hope, my mother is so influential in my life since she gives me hope, thus, I visit my mother frequently, but I must be the source of hope to my husband and sympathizes him because he has the problem.”

**Media**

Informing properly and suitably through media has a great impact on promising to treat infertility and raise knowledge to keep on treatment. A woman with 14-year history of treatment and several failures states: “when I am so distress and depressed hearing some words on T.V or reading a book gives me hope. The information is really effective; for example, it says there is no infertility anymore, all are cured, etc.” Investigating hope decreasing factors, two main themes were observed; one nature of treatments, and the other one negatively oriented mind. These include other subthemes which are pointed out:

**Negatively oriented mind**

**Degradation of wishes and desires**

Most people with infertility problem have negative perspectives, considering all their wills and wishes are lost. Some find negative imaginations and thoughts in frustration so that one of these people says: “I just make myself frustrate by grieving and telling myself that I cannot bear a child. I mean my thoughts make me frustrate, puzzle, and get enough of world. Sometimes I wish I was not born to suffer such pains.” 

**Rough road**

Hardness and toughness in the course of treatment hinder the continuity of treatment. Suffer and roughness as nightmares suppresses and devastates wishes and desires. The effects of these issues are observed in everyday life and performing daily routines so that one expresses that: “as soon as I understood I am infertile, I have less motivation, think more, think about the result of my work, and do not like to do my chores, and I pay attention to my husband less, I found that I have a hard and rugged road ahead,...” 

**Life troubles and difficulties**

A woman with the history of failure says; “I’m absolutely frustrated right now, however, I’m still going on. It's a hard life, an uncertain future ….” 

**Fear and hope**

People who have failed in the treatment of infertility in the range of feelings hopes and fears are gone. It feels a mutual sense of excitement that always threatens their lives." We live in constant fear, we live in hopes and fears that mean trembling of treatment failure"

**Nature of treatments**

Nature of treatment is considered another cause of frustration, and treatment continuity theme in a lot of expressed experiences and themes such as Difficult and painful treatments, Lack of facilities and Expensive treatments have also been named. Treatment effects on physical injuries resulting from it have been observed in the experiences of a lot of people so that the toughness of these treatments and failure in treatment procedure make the people frustrate. Low rate of treatment success, high cost, numerous treatments are also nature of infertility treatments that in many participants it is implicitly referenced.

**Difficult and painful treatments**

One who has a record of failure in IUI treatment mentions: “I don’t visit doctor if IUI doesn’t work this time. Some months ago I took color photo of my uterus and learned that my oviduct is closed; I took a lot of troubles. I pain overnight, I cannot sleep because of ...” 

**Lack of facilities**

Lack of medical facilities has also been mentioned by some others. “Experienced doctors are few in this field; there aren’t expiries in small city. I was told to visit a doctor, but it didn’t work....” 

**Expensive treatments**

Mostly treatment is one of the challenging issues. High cost of medicine on one hand, and failure in treatment procedure on the other hand, decrease the hope in individuals. While crying, another woman states: "I took IUI three times and IVF twice, my husband is a worker, we were told I cannot bear a child, we sold everything for treatment cost, etc."

**Table I:** Emerged themes and sub-themes of increasing and decreasing factors of hope in participants

**Hope increasing factors**	
**Main themes**	**Sub-themes**
Spiritual resources	
	Saying prayer to God
	Family prayer
	Shrines
Family interaction and support	
	Couples' interaction sympathy
Media	Good informing
**Hope decreasing factors**	
Nature of treatments	
	Difficult and painful treatments
	Lack of facilities
	Expensive treatments
Negatively oriented mind	
	Degradation of wishes and desires
	Rough road
	Life troubles and difficulties
	Fear and hope

## Discussion

Aim of this study was live experiences of people from increasing and decreasing factors of hope in infertility treatments. The results of this study revealed that there are three effective factors to increase hope in infertile women as spiritual resources, media, and family sympathy and supports. Hope for infertility treatment is mentioned as one of the effective factors in IVF success (13). This is more important than being pessimist may be negatively affect infertility treatments outcomes (29). Similar to our study, a study conducted by Dafei *et al* demonstrated that 89.4% of the infertile women resort to prayer also remarkable number of them do obligatory or recommended acts and charitable works. Evidence has demonstrated that psychological factors are really important sign and better predictor of biological answers to the treatment than any individual predictors (30). Considering infertility, people who were more decisive in performing religious obligatory acts applied more active coping (28). 

Some studies verify our results considering the effect of increasing hope factors from infertility treatment. The results of present study also revealed that relative supports and close relationship with them are led to increasing hope and motivation to keep on the treatment. Individuals can fight stressors by positive understandings from social supports and high hope which is led to mental health and this psychological well-being is a reaction to facilitation and following the future direction (29). Based on our study, the interaction of family and spouse is effective factor in hope, however, the study of Vassard *et al* demonstrated that social relations were effective in making decision to terminate the fertility (31). 

On one hand, communication difficulties with their partner for men and frequent challenge with their partner for women on the other hand reduced fertility treatment (32). In the identified themes in the present study, media had a remarkable role in promoting hope through informing individuals. It also has been pointed that consultation to infertile women should include both negative and positive aspects, assessment of response to treatment and assisting recognizing the future. It seems that Su has concentrated more on consultation (13). Our study showed that three factors decrease hope in treatment process. These factors contained "Degradation of wishes and desires, rough road, and life troubles and difficulties". In the line with our results, Peddie *et al* reported that women encountered difficulty to accept the fact that their infertility would not be solved (32). 

The success of the treatment at the beginning of infertility treatments were under the pressure of the media and the society. These findings are identical to two subthemes of our study about the treatment of the infertility effects on the complexities of life and their affliction from the treatments Behbahani *et al* demonstrated that women underwent different negative emotional feelings such as loneliness, feeling guilty, depression, and isolation through infertility treatments (33). Another study carried out on the couples who experienced infertility treatment which showed underwent feeling of insufficiency, disappointment, and frustration (34). These results are agreed with our results, are evoke the difficulties of living in infertile couples' life. Fear and hope are factors in decreasing hope in these women. 

In agreement with this result, Imeson and Mc murray showed that couples who underwent ART experienced emotional feeling as an alternative feeling of hope and disappointment inside powerlessness, hope-disappointment cycle and social isolation through life (35). Domar *et al* pointed out that the most typical cause of interruption of treatment is stress (39%) and the basic cause of this stress is the fear of treatment failure. These results agree with our results about decreasing hope in ART (36).

In the present study negatively oriented mind is a factor that decreases hope in patients with ART. In line with the obtained results, Miles, clarified that 42% of the participants underwent high level of general distress through cognitive appraisal (37). Other studies reported that more that 20% of women demonstrate some types of subclinical anxiety or depression six month after the unsuccessful treatment at the follow up. Determinant factors on the course of emotional factors include personality features, definition of fertility complexes, and social supports (38).

Lack of facilities, in the present study, was one of the most important factors that decreases hope in couples undergoing ART. Agree with our study, it was shown that the notion should be elaborated to two more aspects to obtain true high quality ART: timeliness, and patient-centered (39). Regarding patients’ viewpoints over fertility care in a qualitative study, it was demonstrated that fertile patients required medical skills, veneration, coordination, getting acquired about the problem and the treatment, easiness, support, engagement of the partner in the process with positive viewpoint, appropriate relationship with the clinic staff, and ease of access to service (40). According to patients, others believe that insufficient patient-centered was a weak point in fertility care (41). 

Other factors decreasing hope in current study were lack of facilities, difficult and painful treatments, and expensive treatments. In the line with our study, another study capitalized on the mental barriers including financial problems, physical problems, participants’ age, insufficient experience of the health stuff, and treatment cost (1). Overall, infertile patients with infertility treatments need for medical skills, respect, coordination, accessibility, information, comfort, support, partner involvement and a good attitude of and relationship with fertility clinic staff (42). Research limitations were entering to privacy of patients with failure in infertility treatments, need to self-disclosure and express negative feelings. However all of the participants attended to participate in the study and there was no compulsion to participate in the interviews. What is particularly important is recognizing the factors which can provide increase or decrease of individuals’ hope from infertility treatments. This important issue may be help to Policymakers with a comprehensive plan on making optimal use of all the medical facilities for users.

## Conclusion

Applying supportive interventions for the relatives of infertile couples make them change their attitudes toward infertile issue and also sustain their positive relationship with the infertile couples. In this condition, it is expected that their encouragements make the couples keep on the treatment procedure. On the other hand, the role of consultant should not be neglected since the appropriate and systematic consultation helps them to be hopeful and people who are never able to bear a child are led in a direction to accept the current situation to be protected from devastating psychological effects of this problem. 
